# Demonstration of background rates of three conditions of interest for vaccine safety surveillance

**DOI:** 10.1371/journal.pone.0210833

**Published:** 2019-01-15

**Authors:** Anne E. Wormsbecker, Caitlin Johnson, Laura Bourns, Tara Harris, Natasha S. Crowcroft, Shelley L. Deeks

**Affiliations:** 1 St. Joseph’s Health Centre, Toronto, Ontario, Canada; 2 St. Michael’s Hospital, Toronto, Ontario, Canada; 3 Department of Pediatrics, Faculty of Medicine, University of Toronto, Toronto, Ontario, Canada; 4 Public Health Ontario, Toronto, Ontario, Canada; 5 Department of Epidemiology and Community Medicine, University of Ottawa, Ottawa, Ontario, Canada; 6 Department of Laboratory Medicine and Pathobiology, University of Toronto, Toronto, Ontario, Canada; 7 Dalla Lana School of Public Health, University of Toronto, Toronto, Ontario, Canada; Uniformed Services University of the Health Sciences, UNITED STATES

## Abstract

**Introduction:**

Adverse events following immunization (AEFIs) are unwanted or unexpected health outcomes following vaccination, which may or may not be causally-linked to vaccines. AEFI reporting is important to post-marketing vaccine safety surveillance and has the potential to identify new or rare AEFIs, show increases in known AEFIs, and help to maintain public confidence in vaccine programs. Knowledge of the expected incidence (i.e. background rate) of a possible AEFI is essential to the investigation of vaccine safety signals. We selected three rarely reported AEFIs representing the spectrum of causal association with vaccines, from proven (immune thrombocytopenia [ITP]) to questioned (Kawasaki disease [KD]) to unsubstantiated (multiple sclerosis [MS]) and determined their background rates.

**Methods:**

We extracted data on hospitalizations (CIHI Discharge Abstract Database) for ITP, KD, and MS among Ontario children for the period 2005 to 2014 from IntelliHEALTH. As ITP can be managed without hospitalization, we also extracted emergency department (ED) visits from the CIHI National Ambulatory Care Reporting System. For all conditions, we only counted the first visit and if the same child had both an ED visit and a hospitalization for ITP, only the hospitalization was included. We calculated rates by year, age group and sex using population estimates from 2005–2014, focusing on age groups within the Ontario immunization schedule around vaccine(s) of interest.

**Results:**

Per 100,000 population, annual age-specific incidence of ITP in children age 1 to 7 years ranged from 8.9 to 12.2 and annual incidence of KD in children less than 5 years ranged from 19.1 to 32.1. Average annualized incidence of adolescent (11–17 years) MS across the study period was 0.8 per 100,000.

**Discussion:**

Despite limitations, including lack of clinical validation, this study provides an example of how health administrative data can be used to determine background rates which may assist with interpretation of passive vaccine safety surveillance.

## Introduction

Monitoring for potential adverse events following immunization (AEFI), whether they are in relation to new vaccines, immunization schedule changes, or existing vaccines, is the core of post-marketing vaccine safety surveillance. In the Canadian province of Ontario, AEFI reporting by designated health care providers is mandatory under the Health Promotion and Protection Act 1990; however, vaccine recipients or their caregivers can also voluntarily report AEFI. With the exception of active surveillance at two children’s hospitals, most vaccine safety monitoring is through passive surveillance. A full description of AEFI surveillance in Ontario has been published elsewhere [1]. We undertook the present analysis in the context of Ontario’s broader efforts, including revised case definitions and the publication of an annual report [1], to strengthen its vaccine safety surveillance.

The background rate of diseases that could be AEFI can be used to estimate the number of expected events within a known population over a period of time. When AEFI are reported, further investigation may be warranted if the number of observed events is greater than would normally be expected based on the background rate of the condition of interest. Knowledge of background rates are especially useful when a new vaccine is introduced or a schedule change is made. Several studies during and following the 2009–10 H1N1 pandemic addressed the importance of understanding the existing background rates of relevant rare and serious medical conditions that could be reported as AEFI (e.g. Guillain-Barre syndrome [GBS] after influenza immunization) when assessing the safety of a mass vaccination campaign [2–4].

We used health care administrative data to explore Ontario’s background rates of three rarely reported AEFI in childhood that have the potential to raise concerns in relation to current or future immunization programs. The three conditions, immune thrombocytopenia (ITP), Kawasaki disease (KD) and multiple sclerosis (MS), respectively, represent a range of causal association with immunization–from proven, to questioned, to unsubstantiated. Thrombocytopenia has a proven causal relationship with measles-containing vaccine [5–8] and, to a lesser degree, other vaccines [9]. The median estimated incidence of thrombocytopenia due to the measles, mumps, rubella (MMR) vaccine is 2.6 cases per 100,000 doses (range 0.09 to 4 cases per 100,000 doses) [10]. It normally occurs within 6 weeks after vaccination [7] and is more likely to occur after the first dose [11]. Ontario’s publicly-funded vaccination program includes MMR vaccine at one year and measles-mumps-rubella-varicella (MMRV) vaccine at 4–6 years [12].

KD has been consistently reported in passive paediatric AEFI surveillance [1,13,14]. Given the proposed role of an immunologic response in the unknown etiology of this acquired systemic childhood vasculitis, immunization has been questioned as a possible cause [15,16], but a recent systematic review found no association between KD and immunization [17]. Of note, active hospital-based surveillance of KD in Ontario has shown that the incidence of KD in Ontario may be one of the highest outside of Asia [18].

Unsubstantiated hypotheses drawing public, media, and even legal, attention [19,20] have been raised that immunization, specifically hepatitis B and HPV vaccines [21,22], may have an association with multiple sclerosis (MS), but numerous studies and reports have found no causal relationship [23,24]. In Ontario, hepatitis B vaccine is given in Grade 7 (age approximately 12 years) and human papillomavirus (HPV) vaccine was initially given to Grade 8 females beginning in 2007, with the addition of males and change to Grade 7 in 2016.

Our study had three objectives. The first was to determine the rates of ITP, KD and MS in AEFI-relevant age groups and the second was to demonstrate that appropriate background rates can be found from readily available Ontario health care administrative data. Finally, we aimed to demonstrate the use of background rates by applying our findings to a hypothetical vaccinated cohort to determine an expected number of AEFI.

## Materials and methods

Ontario is Canada’s largest province (population ~14 million, 2016 [25]) and has a single-payer, publicly-funded health care system. Provincial health care administrative data are collected by the Canadian Institute for Health Information (CIHI) and provided to the Ministry of Health and Long-Term Care (MOHLTC). CIHI data are included in the MOHLTC’s IntelliHEALTH knowledge repository, which contains de-identified data collected from various sectors of Ontario’s health care system. Public health, health care and government analysts, epidemiologists, planners, policy and decision makers, and researchers have secure online access to IntelliHEALTH. This access is provided through the MOHLTC’s information access policies, based on the Personal Health Information Protection Act. Of note, although IntelliHEALTH is readily available to local public health agencies, it does not contain immunization information.

While the Brighton Collaboration refers to low platelets after immunization simply as thrombocytopenia (platelets less than 150 x 10^9^/L) rather than ITP, to differentiate vaccine-associated thrombocytopenia from that with unknown etiology [10], we studied ITP as a case would likely be recorded as ITP in health care administrative data prior to making an association with a vaccine. KD and MS were defined based on International Classification of Diseases (ICD-10) diagnostic codes in administrative data. We were not able to separate KD cases into definite, probable and possible KD in the manner of the Brighton Collaboration [17]. See S1 Table for ICD-10 codes used for each medical condition.

We carried out descriptive analyses of case counts and rates over time. Incidence of health care utilization for ITP, KD and MS in relevant age groups were calculated using data from CIHI’s Discharge Abstract Database (CIHI-DAD Most Responsible Diagnosis [MRDx] codes) and the National Ambulatory Care Reporting System (CIHI-NACRS), containing information about hospitalization and emergency department (ED) visits, respectively.

We extracted data for these three conditions on February 5, 2016 and included the time period from January 1, 2005 to December 31, 2014. We limited our population to children and adolescents, and specifically to age groups that reflect Ontario’s routine, publicly-funded immunization schedule [12] for vaccines questioned to have an association with each of our selected conditions. For ITP, we assessed children aged 1–7 years as both scheduled measles-containing vaccine doses should be given by then; for KD we studied all children 0–9 years, based on age-related risk of KD since there is no specific vaccine associated with this condition; and for MS we focused on adolescents 11–17 years as the school-based hepatitis B and HPV vaccines are given within the early part of this age band.

We only considered individuals once for each condition, in an attempt to separate incident events from repeat health care encounters for the same illness. For ITP, we included both hospitalized individuals and those who had an ED visit because ITP can be managed without hospitalization, however if they were present in both data sources, we only counted the hospitalization. We only included cases of KD and MS that were hospitalized given the likely need for hospitalization to make diagnosis and provide treatment at the time of first presentation in children. We also used the ITP incidence from our analysis to explore the expected number of AEFI cases given estimated MMR coverage and the associated ITP risk from the literature.

## Results

Age-specific counts and incidences of health care utilization for ITP, KD and MS (also sex-specific) are shown for the age groups of interest in Table 1 for the study period overall.

**Table 1:** Counts and incidence of immune thrombocytopenia, Kawasaki disease and multiple sclerosis among Ontario children, 2005–2014.

**Condition**	**Age Group**		**n**	**Incidence per 100,000 persons**
**Immune thrombocytopenia**	1–7 years		1,053	10.5
**Kawasaki disease**	<5 years		1,606	22.8
	5–9 years		516	6.7
	All (0–9 years)		2,122	14.4
**Multiple sclerosis**	11–17 years	Female	66	1.2
		Male	22	0.37
		Total	88	0.8

**Fig 1:**
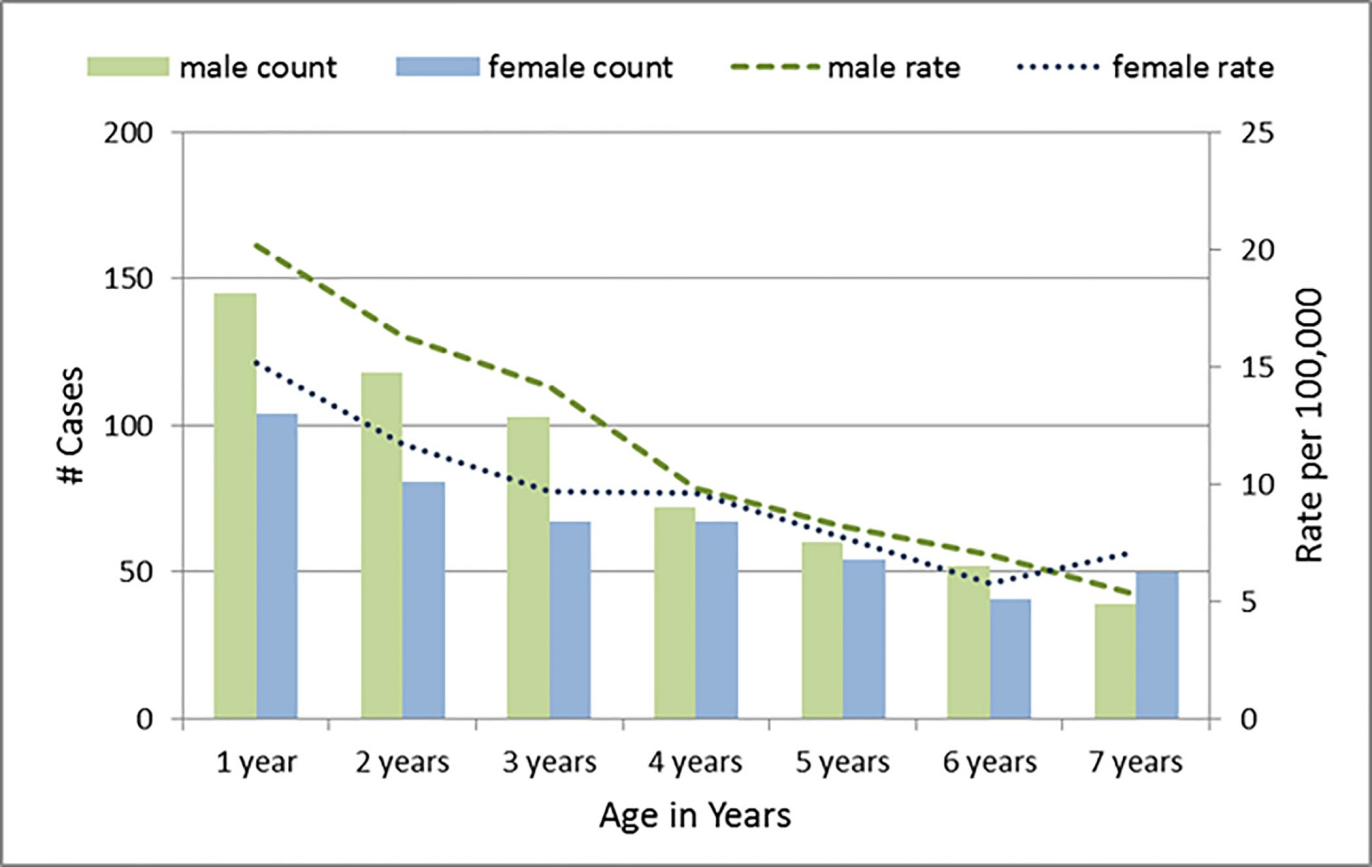
Immune thrombocytopenia hospitalizations and emergency department visits among Ontario children age 1–7 years, by age and sex, 2005–2014.

A total of 1,053 children age 1 to 7 years received a diagnosis of ITP during the 10-year study period. The majority of these (n = 804, 76%) were hospitalized. Annual incidence rate of ITP was relatively constant; varying slightly between 8.9 per 100,000 (2013) and 12.2 per 100,000 (2012). The incidence of ITP was highest among children aged 1 year (17.6 per 100,000) and decreased in subsequent ages. Boys aged 1 year had the highest incidence of any group (20.1 per 100,000). Higher rates of ITP were observed in boys versus girls up to the age of 6 years, with the male to female ratio decreasing as age increased. At seven years of age, the female rate was higher than that of males (Fig 1).

**Fig 2:**
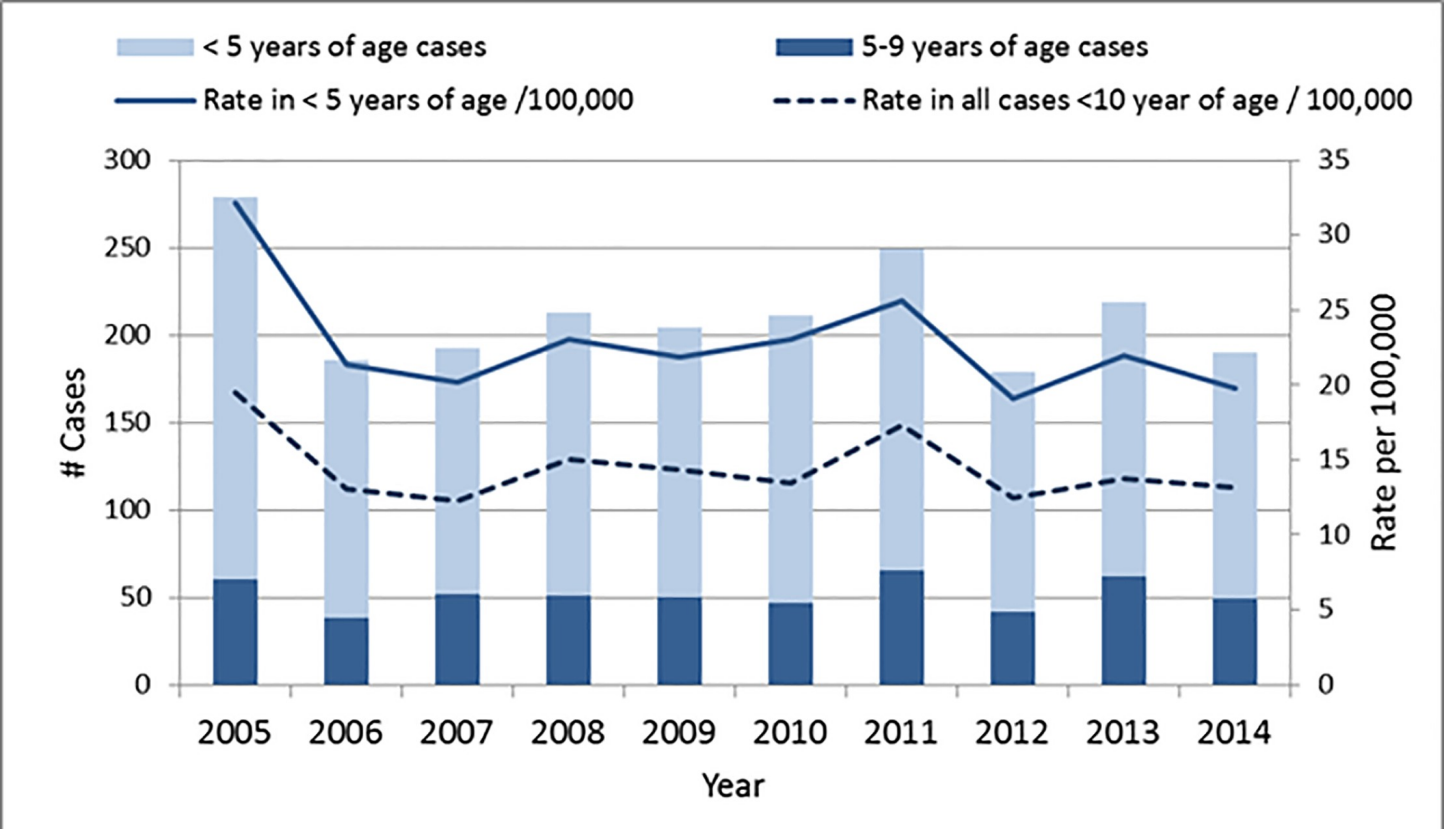
Kawasaki disease hospitalizations among Ontario children age 0–9 years, by year, 2005–2014.

**Fig 3:**
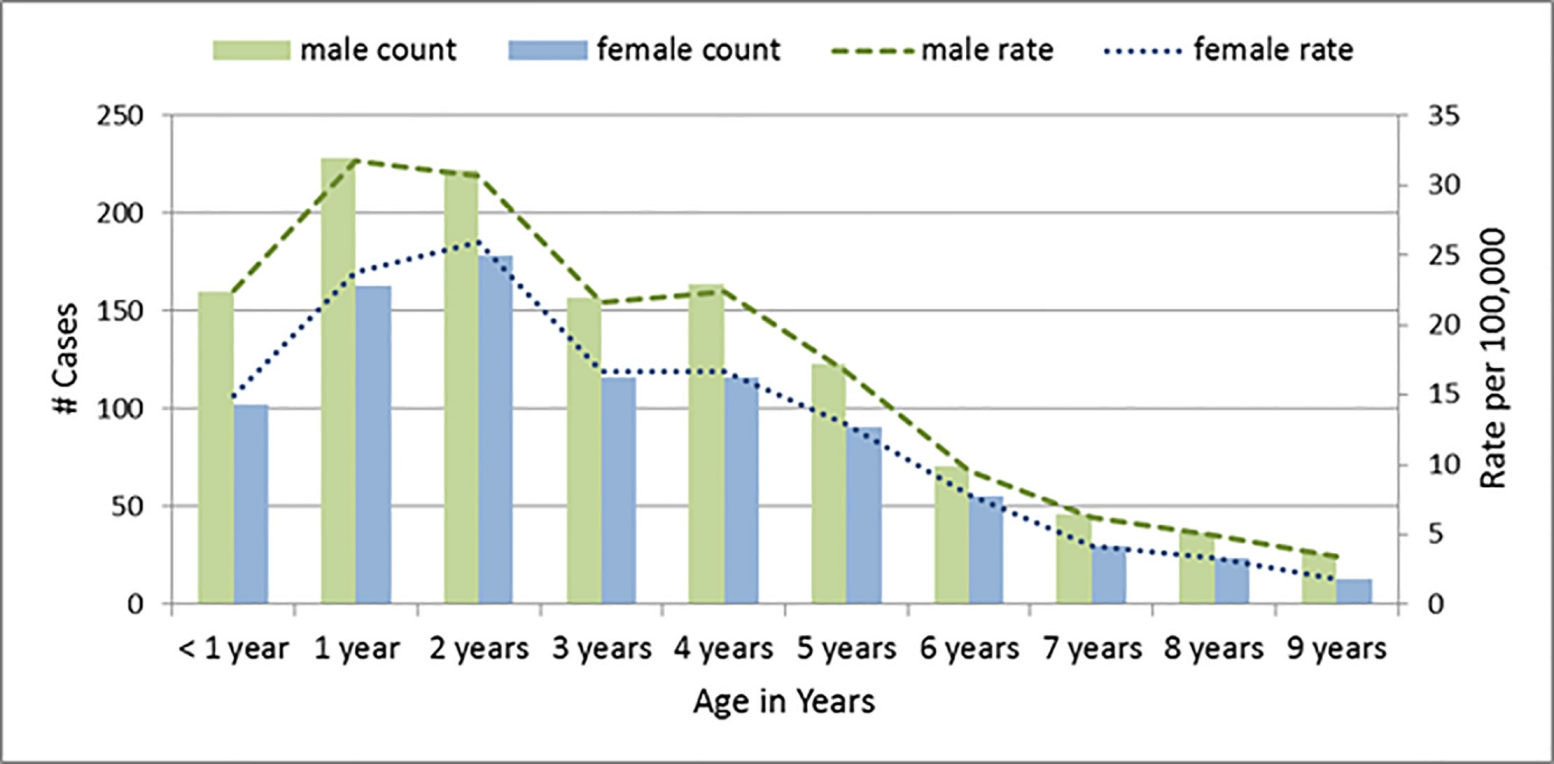
Kawasaki disease hospitalizations among Ontario children age 0–9 years, by age and sex, 2005–2014.

A total of 2,122 children less than 10 years were hospitalized with a diagnosis of KD during the study period. The average annual incidence rate of KD in this age group was 14.4 per 100,000 for the entire study period. As displayed in Fig 2, the annual incidence varied between a low of 12.3 per 100,000 (2007) and a high of 19.5 per 100,000 (2005). When the age group was restricted to younger children, rates were higher. The average annual incidence among children less than 5 years was 22.7 per 100,000 children and ranged from a low of 19.1 (2012) to a high of 32.1 (2005) per 100,000. When assessed by age and sex, the rate of KD peaked at 1–2 years of age and decreased in subsequent age groups (Fig 3). Boys aged 1 and 2 years had the highest incidence at 31.7 and 30.7 per 100,000, respectively. Higher rates of KD were seen in males, with the male to female ratio decreasing from infancy into older childhood (Fig 3).

The total number of adolescent (age 11–17 years) MS hospitalizations in Ontario from 2005 to 2014 was very low (n = 88), and the average annualized incidence rate across the study period was 0.8 per 100,000 adolescents (Table 1). We found three times as many cases in girls (n = 66) than boys (n = 22).

To demonstrate how these findings are applicable to public health, we used the known risk of ITP following MMR immunization and immunization coverage of a population to determine the number of cases of ITP attributable to the first dose of MMR. The risk of ITP after MMR vaccine has been estimated as 2.6 cases per 100,000 doses [10]. The Ontario annual birth cohort is approximately 140,000, with an estimated 96% MMR coverage [26], (based on one dose rubella coverage as Ontario vaccination coverage is reported by antigen and measles and mumps coverage are only assessed after two doses). Thus, approximately 134,400 one-year-olds in Ontario are vaccinated with MMR each year. If the risk of ITP following MMR vaccine is applied to these 134,400 children, we would expect 3.5 cases of ITP each year to be attributable to the first dose of MMR. We found an average of 25 cases per year of ITP in children age one year, which means approximately 7% (3.5/ 25) of ITP cases in 1 year old children in Ontario could be due to MMR vaccine, using the risk estimate from the literature.

## Discussion

The background rates we have estimated for ITP and KD are consistent with rates obtained through different methods in the same or similar settings, but we were not able to draw this conclusion regarding MS. With the caveat that caution should be used when comparing rates across literature and data sources, methods of analysis, health care systems and populations, other studies report similar incidence of childhood ITP to ours. We were unable to find comparable Ontario or Canadian data on the incidence of ITP in children, but a United Kingdom study using administrative data from 1990 to 2005 reported a rate of ITP of 6.8 per 100,000 person years in children <2 years and 7.2 per 100,000 person years in those aged 2–5 years [27]. A systematic review of ITP incidence among a broader age group of children in several European countries reported the incidence of ITP to be between 1.9–6.4 cases per 100,000 children < 16 years of age [28]. The single year prevalence of ITP (>2 ITP insurance claims separated by at least 30 days) in 2002 in the American state of Maryland was 9.3 per 100,000 children aged 1–5 years [29]. Although the setting, methods, and age groups were different, this finding is similar to the incidence we found (10.5 per 100,000 among children 1–7 years) for this acute condition. The sex distribution of pediatric ITP we observed was also similar in other studies, with the majority of cases being male, especially in the younger age groups [27,29–30].

Other investigators have validated the use of administrative data to determine the incidence of KD by comparing administrative data results to active surveillance mechanisms in Ontario [31]. Further, a review of hospitalization data estimated the annual incidence of KD in Ontario to be between 24–27 cases per 100,000 children < 5 years between 2004 and 2011 [32]. Our finding of 22.8 cases per 100,000 children <5 years is comparable with the slightly lower rate possibly owing to the different time period assessed and our more limited inclusion criteria (only most responsible diagnosis versus primary or secondary diagnosis codes [32]). Our data showed higher rates of KD in males, especially in the younger ages (i.e. less than 5 years of age), which is also consistently found in the literature [18,33–36].

As pediatric MS is rare, and can manifest differently than adult MS, the incidence and prevalence of adolescent MS is not well known. However, the Atlas of Multiple Sclerosis, a collaboration between the Multiple Sclerosis International Federation (MSIF) and the World Health Organization (WHO) estimated the 2013 prevalence of pediatric MS in Canada to be between 0.52–0.56 per 100,000 children [37]. Our calculated incidence rate (average of 0.8 per 100,000 adolescents aged 11–17 years) is higher, but we limited the denominator to a higher-risk age group (adolescents) and did not exclude those who had MS hospitalizations prior to 2005. On the other hand, our result could also be an underestimate, as we did not include outpatient visits. A recent validation of the use of Ontario administrative data in estimating the incidence of MS in the adult population found that optimal sensitivity (84%) and positive predictive value (86%) required one hospitalization or five physician office billings over 2 years [38]. It is unknown whether this algorithm would perform as well for adolescents, but highlights a limitation of our data, which did not include physician billings.

While we are pleased to have demonstrated that background rates can be estimated from intelliHEALTH, a readily available dataset, we are aware of the limitations of our study. We did not undertake a chart review to validate the diagnoses obtained from health care administrative data. Further, individuals who did not seek health care at all and those who did not attend a hospital (i.e. physician office visits only) are not included. As with any uncommon condition, rates can be unstable with small numerators, which would cause our results to be inaccurate. This concern is mitigated by consistent case definitions and monitoring temporal trends rather than rates *per se*. More broadly, there is a 3–6 month time lag in CIHI data availability through intelliHEALTH. This could limit its use in timely assessment of vaccine safety signals, which usually require a rapid response.

Limitations notwithstanding, the background rates we calculated also serve as a point of comparison against incidence of these conditions in our provincial AEFI surveillance. “Locally-relevant” background rates are an important component of vaccine safety surveillance [2]. Based on the provincial case definition for thrombocytopenia in Ontario’s AEFI surveillance, there was one case of thrombocytopenia in a child in 2015 [14] and there were three in 2016 [38]. None of the cases of thrombocytopenia had received measles-containing vaccine (Public Health Ontario, unpublished data) despite the established link and the number predicted in our calculations above (3.5 cases of ITP expected in children aged one year after MMR vaccine). The lack of MMR-associated ITP cases in recent Ontario AEFI reports [1,14,39], suggests under-reporting.

No case definitions exist for KD or MS in routine Ontario AEFI surveillance [40], but these conditions would be captured if reported as “other severe or unusual events”. Searching the 2015 and 2016 provincial vaccine safety data by key terms, there were five cases of children with KD and no cases of adolescent MS [41].

Calculation of these background rates serves to further strengthen our provincial vaccine safety surveillance system, which has been gaining momentum in recent years with increasing availability and quality of vaccine safety data [1,14,41], and recognition of Public Health Ontario’s work by the World Health Organization [42]. We are well positioned to rapidly assess safety reports related to these three conditions, comparing them to known background rates and to evaluate the sensitivity of the system to be able to capture reportable events. With this study as an example, we and others in Canada could consider the use of intelliHEALTH, or similar sources of administrative data, in future to determine background rates of conditions reported as AEFI.

## Supporting information

S1 Table ICD-10 most responsible diagnosis codes used to identify disease outcomes. (DOCX)
