# Supplementary material for: Demonstration of background rates of three conditions of interest for vaccine safety surveillance
Source: PLoS One. 2019 Jan 15;14(1):e0210833. doi: 10.1371/journal.pone.0210833 (PMC6333343; doi:10.1371/journal.pone.0210833)
Supplement: S1 Table — (DOCX) [file pone.0210833.s001.docx]

Childhood vaccine safety: background rates of three conditions of interest

Anne E. Wormsbecker, Caitlin Johnson, Laura Bourns, Tara Harris, Natasha S. Crowcroft, Shelley L. Deeks

**Supporting Information**

**S1 Table: ICD-10 most responsible diagnosis codes used to identify disease outcomes**

| Condition | Data Source | ICD-10 codes included | | ICD-10 codes excluded | |
| --- | --- | --- | --- | --- | --- |
| Immune thrombocytopenia | CIHI-DAD and CIHI-NACRS | D69.3 | Idiopathic thrombocytopenic pupura | Q87.2 | Thrombocytopenia with absent radius |
|  |  | D69.38 | Other idiopathic thrombocytopenic purpura | P61.0 | Transient neonatal thrombocytopenia |
|  |  | D69.4 | Other primary thrombocytopenia | D82.0 | Wiskott-Aldrich syndrome |
|  |  | D69.6 | Thrombocytopenia, unspecified | C81-C96 | Malignant neoplasms of lymphoid, haematopoietic and related tissue |
|  |  |  |  | D46, D46.0-D46.9 | Myelodysplastic syndromes |
| Kawasaki disease | CIHI-DAD | M30.3 | Mucocutaneous Lymph node syndrome (Kawasaki disease) | --- | |
| Multiple sclerosis | CIHI-DAD | G35 | Multiple sclerosis (of): NOS, brain stem, cord, disseminated, generalized | --- | |
